# Oral Tau Aggregation Inhibitor for Alzheimer’s Disease: Design, Progress and Basis for Selection of the 16 mg/day Dose in a Phase 3, Randomized, Placebo-Controlled Trial of Hydromethylthionine Mesylate

**DOI:** 10.14283/jpad.2022.63

**Published:** 2022-06-26

**Authors:** Claude M. Wischik, P. Bentham, S. Gauthier, S. Miller, K. Kook, B. O. Schelter

**Affiliations:** 1 TauRx Therapeutics Ltd, 395 King Street, Aberdeen, AB24 5RP UK; 2 School of Medicine, Medical Sciences and Nutrition, University of Aberdeen, Foresterhill, Aberdeen, UK; 3 Alzheimer’s Disease Research Unit, McGill Centre for Studies in Aging, Montreal, Canada; 4 Salamandra LLC, Bethesda, Maryland USA; 5 Institute for Complex Systems and Mathematical Biology, University of Aberdeen, Aberdeen, UK

**Keywords:** Leuco-methylthioninium bis(hydromethanesulphonate), LMTM, hydromethylthionine mesylate, Alzheimer’s disease, tau aggregation inhibitor

## Abstract

**Background:**

Hydromethylthionine mesylate is a tau aggregation inhibitor shown to have exposure-dependent pharmacological activity on cognitive decline and brain atrophy in two completed Phase 3 trials in mild/moderate Alzheimer’s disease (AD).

**Objectives:**

The present report summarises the basis for selection of 16 mg/day as monotherapy as the optimal treatment regime and the design rationale of a confirmatory Phase 3 trial (LUCIDITY).

**Design:**

The trial comprises a 12-month double-blind, placebo-controlled phase followed by a 12-month modified delayed-start open-label treatment phase.

**Setting:**

76 clinical research sites in North America and Europe.

**Participants:**

545 patients with probable AD or MCI-AD in the final version of the protocol.

**Intervention:**

Participants were assigned randomly to receive hydromethylthione mesylate at doses of 16 mg/day, 8 mg/day or placebo at a 4:1:4 ratio during the double-blind phase. All participants in the open-label phase receive the 16 mg/day dose.

**Measurements:**

Co-primary clinical outcomes are the 11-item Alzheimer’s Disease Assessment Scale (ADAS-cog_11_) and the 23-item Alzheimer’s Disease Cooperative Study — Activities of Daily Living (ADCS-ADL_23_). Secondary biomarker measures include whole-brain atrophy and temporal lobe ^18^F-fluorodeoxyglucose positron emission tomography.

**Results:**

446 participants are expected to complete the 12-month placebo-controlled phase in March 2022.

**Conclusions:**

If the primary end points are met, the data will provide confirmatory evidence of the clinical and biomarker benefits of hydromethylthionine mesylate in minimal to moderate AD. As low-dose oral hydromethylthionine mesylate is simple to use clinically, does not cause amyloid-related imaging abnormalities and has a benign safety profile, it would likely improve AD management.

## Introduction

**T**here is an urgent need for a disease-modifying treatment for Alzheimer’s disease (AD) that could be used both for treatment of clinical stages of dementia, as well as early prevention (1, 2). In the United States, 6.2 million adults aged over 65 years have AD dementia, and the annual healthcare payments for AD are expected to increase from $355 billion in 2021 to more than $1.1 trillion in 2050 (3). To reduce the global socio-economic burden meaningfully, AD treatments need to be safe, clinically effective, and widely accessible (4). The available treatments (acetylcholinesterase inhibitors [AChEIs] and the NMDA receptor antagonist memantine) provide only temporary relief from cognitive and behavioral symptoms. Although aducanumab (Aduhelm) has the potential to modify the underlying disease by targeting amyloid, it has not been approved outside of the USA and UAE (5, 6). Aducanumab is associated with a high risk of amyloid-related imaging abnormalities (7), need for monthly intravenous infusions and repeated magnetic resonance imaging (MRI) monitoring, and there is a lack of consensus on interpretation of clinical efficacy findings (8–10). The major clinical and societal needs, therefore, remain largely unmet.

Tau aggregation is a promising target for disease-modifying AD therapy as it is a hallmark pathology of AD that correlates with AD clinical severity and progression more strongly than amyloid pathology (11, 12). TauRx Therapeutics has been developing an orally administered tau aggregation inhibitor (TAI), hydromethylthionine mesylate (the international nonproprietary name for leucomethylthionium bis(hydromethanesulphonate), LMTM). Hydromethylthionine mesylate is a stable crystalline form of hydromethylthionine, the reduced form of methylthionine (MT; (13)). Hydromethylthionine reduced the tau pathology and behavioural impairments in tau transgenic mouse models (14). It acts both by inhibiting tau aggregation (15) and by disaggregating pathological tau oligomers and filaments (13, 16). Phase 2 and 3 trials demonstrated a benign safety profile (19, 20). This article describes the basis for selection of the 16 mg/day dose and the design rationale of the ongoing Phase 3 clinical trial which aims to confirm the safety and efficacy of hydromethylthionine mesylate in AD.

### Basis for selection of the 16 mg/day dose of hydromethylthionine mesylate

Clinical development of the MT moiety began with methylthioninium chloride (MTC, commonly known as methylene blue) (17). In a Phase 2 dose-finding clinical trial in mild to moderate AD a dose of 138 mg/day of MT given as MTC reduced cognitive and functional brain metabolic decline (17). Subsequently, hydromethylthionine mesylate, a stable reduced form of MT, was developed to avoid the dose-dependent absorption limitations of MTC (18).

**Figure 1:**
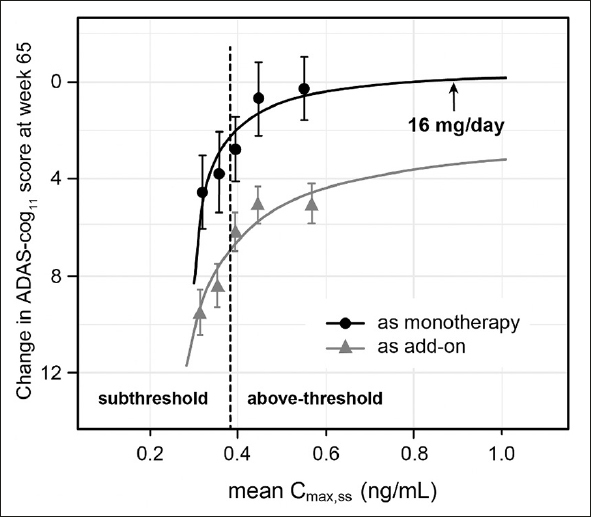
Relationship between steady-state plasma levels of hydromethylthionine and decline on the ADAS-Cog_11_ scale over 65 weeks in 566 participants receiving hydromethylthionine mesylate at 8 mg/day (100 as monotherapy, 466 as add-on to standard AD symptomatic drugs) in completed Phase 3 trials TRx-237-015 and TRx-237-005) Patient groups with subthreshold and above-threshold exposure to hydromethylthionine mesylate 8 mg/day were defined based on the lower calibration limit of the assay (0.373 ng/mL, dotted line). The predicted mean Cmax, ss and change in ADAS-cog_11_ for participants receiving hydromethylthionine mesylate 16 mg/day is shown, based on pharmacokinetic modelling described previously (18). AD = Alzheimer’s Disease; ADAS-Cog_11_ = Alzheimer’s Disease Assessment Scale-Cognitive subscale (11-item); Cmax, ss = steady state maximum plasma concentration.

Hydromethylthionine mesylate has been tested in two completed Phase 3 trials in AD, designed on the basis of the results of the Phase 2 trial (19, 20). It was assumed that a dose of at least 138 mg/day was required for clinical pharmacological activity. Therefore, the active arms received 150–250 mg/day hydromethylthionine mesylate. When MT is excreted, it can discolour the urine. To maintain blinding, the control arm received a low dose of 8 mg/day which was assumed to be clinically inactive. There were no overall differences in the clinical or neuroimaging outcomes between the high and low doses of hydromethylthionine mesylate as randomized. However, a posthoc pharmacokinetic analysis revealed unexpected, exposure-dependent pharmacological activity on key clinical and neuroimaging end points in participants receiving 8 mg/day hydromethylthionine mesylate (21). There were steep exposure-response relationships at the 8 mg/day dose for both cognitive benefit (Figure 1) and rate of progression of brain atrophy. A dose of 16 mg/day was identified as the minimum at which all participants were predicted to have steady-state maximum concentration levels above a threshold identified as being required for clinical benefit in all patients (0.373 ng/mL).

The completed Phase 3 trials also revealed significant differences between participants receiving hydromethylthionine mesylate as monotherapy when compared with those receiving the drug as an add-on to standard symptomatic treatments for AD (AChEIs and/or memantine). These differences in outcome could not be explained by differences in clinical, neuroimaging, or demographic characteristics at baseline in either prespecified (20) or posthoc (19) analyses. Several preclinical studies (22, 23) have now confirmed that the interference in hydromethylthionine mesylate activity produced by the symptomatic AD drugs can be reproduced in a tau transgenic mouse model. These studies provide a neuropharmacological mechanism for the difference in the clinical response to hydromethylthionine mesylate given alone or as an add-on. Specifically, they suggest that the chronic brain stimulation produced by symptomatic drugs results in a compensatory homeostatic downregulation in multiple neuronal systems, leading to a reduction in many of the pharmacological effects of hydromethylthionine mesylate in the brain (22, 23).

**Figure 2:**
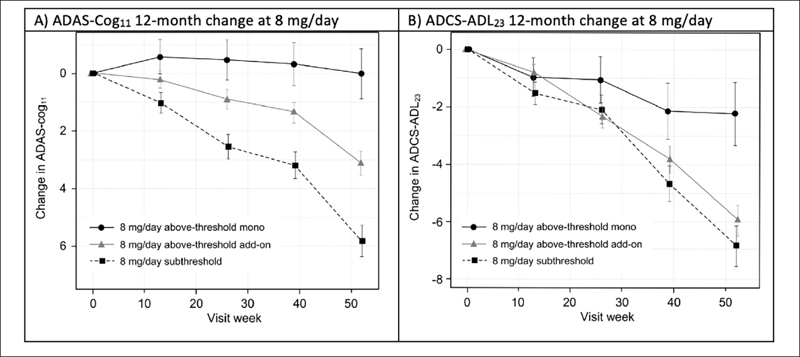
Change in (A) ADAS-Cog_11_ and (B) ADCS-ADL_23_ over 52 weeks in participants receiving hydromethylthionine mesylate at 8 mg/day as monotherapy or as add-on to standard AD symptomatic drugs in completed Phase 3 trials TRx-237-015 and TRx-237-005, grouped according to subthreshold or above-threshold exposure at the 8 mg/day dose Of participants receiving 8 mg/day, 193 had subthreshold exposure and 373 had above-threshold exposure, of whom 67 were receiving HMTM as monotherapy and 306 were receiving HMTM as add-on to standard AD symptomatic drugs in completed Phase 3 trials TRx-237-015 and TRx-237-005; The statistical analysis was as described previously (18). Error bars represent standard error of the mean. AD = Alzheimer’s Disease; ADAS-Cog_11_ = Alzheimer’s Disease Assessment Scale-Cognitive subscale (11-item); ADCS-ADL_23_ = Alzheimer’s Disease Cooperative Study — Activities of Daily Living (23-item).

To permit posthoc evaluation of the clinical effects of hydromethylthionine mesylate according to exposure, participants receiving 8 mg/day hydromethylthionine mesylate were categorized as having steady-state plasma levels of the drug either above or below the threshold of 0.373 ng/mL. Patients with above-threshold exposure to hydromethylthionine were further divided according their co-medication status with the standard symptomatic treatments for AD. Figure 2 shows changes in cognition, measured by Alzheimer’s Disease Assessment Scale-Cognitive Subscale (ADAS-Cog_11_ (24)) and function, measured by Alzheimer’s Disease Cooperative Study-Activities of Daily Living (ADCS-ADL_23_ (25)), over 12 months in participants receiving the 8 mg/day dose. Patients with above-threshold exposure to hydromethylthionine as monotherapy had significantly less decline in cognition and function, compared with participants with subthreshold exposure. Patients with subthreshold exposure to the drug demonstrated a cognitive decline of 5.80 (±0.55) units and functional decline of -6.89 (±0.72) units. In comparison, participants with above-threshold exposure to hydromethylthionine as monotherapy had −5.77 (±1.01) units less cognitive decline (p<0.001) and 4.64 (±1.33) units less functional decline (p<0.001), corresponding to 100% and 67% reductions in decline respectively. Both these findings exceed the differences that are considered clinically meaningful: a 3-unit change on ADAS-Cog and a 2-point change on ADCS-ADL (26, 27). As an add-on to symptomatic treatments, the treatment difference on the ADAS-Cog_11_ scale was reduced by half (−2.68 ± 0.69, p<0.001) and was not significant on the functional scale. Presence of the apolipoprotein E4 allele had no effect on either decline or on the treatment effect.

**Figure 3:**
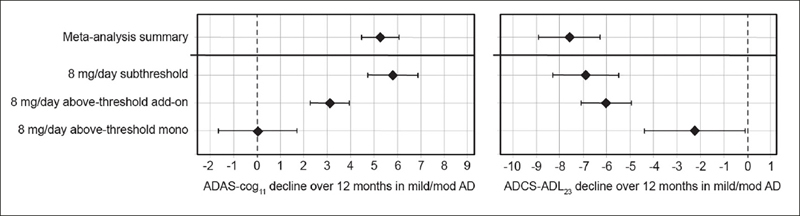
Change in ADAS-Cog_11_ and ADCS-ADL_23_ over 52 weeks in participants receiving hydromethylthionine mesylate at 8 mg/day as monotherapy or as add-on to standard AD symptomatic drugs in completed Phase 3 trials TRx-237-015 and TRx-237-005 according to subthreshold or above-threshold exposure at the 8 mg/day dose compared with the weighted mean placebo decline over 52 weeks, reported in a meta-analysis of studies in mild/moderate AD Of participants receiving 8 mg/day, 193 had subthreshold exposure and 373 had above-thresheold exposure, of whom 67 were receiving HMTM as monotherapy and 306 were receiving HMTM as add-on to standard AD symptomatic drugs. The statistical analysis model was as described previously (18). Error bars represent standard error of the mean. AD = Alzheimer’s Disease; ADAS-Cog_n_ = Alzheimer’s Disease Assessment Scale-Cognitive subscale (11-item); ADCS-ADL_23_ = Alzheimer’s Disease Cooperative Study — Activities of Daily Living (23-item).

Given the unexpected clinical activity of hydromethylthionine at the 8 mg/day dose, the completed Phase 3 trials did not have a true placebo group for evaluation of efficacy. Therefore, a meta-analysis was conducted to determine the clinical decline expected in similar populations participating in placebo groups or observational studies. ADAS-Cog_11_ data were available from 12 data sets reported since 1996 in a total of 4,375 participants. Cognitive decline ranged from 2.90 to 7.93 units, with a weighted mean of 5.26 units (95% CI 4.45, 6.06). For ADCS-ADL_23_, data were available from 5 studies reported between 2012–2019 in a total of 1,296 participants. Functional decline ranged from −5.70 to −10.09 units with a weighted mean of −7.59 units (95%CI, −8.90, −6.29). Comparisons with exposure-dependent responses to hydromethylthionine mesylate are shown in Figure 3. As expected, the cognitive and functional decline in participants with subthreshold exposure to hydromethylthionine at the 8 mg/day dose did not differ significantly from that expected in similar populations in the meta-analysis. Patients with above-threshold exposure to hydromethylthionine as monotherapy showed significantly less decline than that observed historically on both scales (ADAS-Cog_11_ difference −5.23 ± 0.953 units, p<0.001; ADCS-ADL_23_ difference 5.33 ± 1.29 units, p<0.001). Patients with above-threshold exposure to hydromethylthionine as add-on therapy also declined significantly less compared to placebo on the ADAS-Cog_11_ scale (difference −2.14 ± 0.587, p<0.001) whereas on the ADCS-ADL_23_ scale, the trend was directionally similar but not statistically significant (difference 1.56 ± 0.867, p=0.072).

These results have informed the size, duration, and statistical design of the ongoing Phase 3 LUCIDITY trial, which was designed to confirm the safety and efficacy of 16 mg/day hydromethylthionine mesylate monotherapy compared with placebo in participants with minimal to moderate AD.

## Methods

### Study Design

The LUCIDITY trial (NCT03446001; EudraCT: 2017-003558-17) is a Phase 3, randomized, double-blind, placebo-controlled, outpatient trial to evaluate the safety, efficacy, and tolerability of hydromethylthionine mesylate monotherapy in participants with severity ranging from mild cognitive impairment (MCI) to moderate AD. The initial 12-month blinded period is followed by a 12-month, open-label extension period to provide comparative, delayed-start data. There are 76 study sites located in Canada, France, Italy, Poland, Spain, United Kingdom and United States. The protocol was approved by an institutional review board or independent ethics committee at each site.

**Figure 4:**
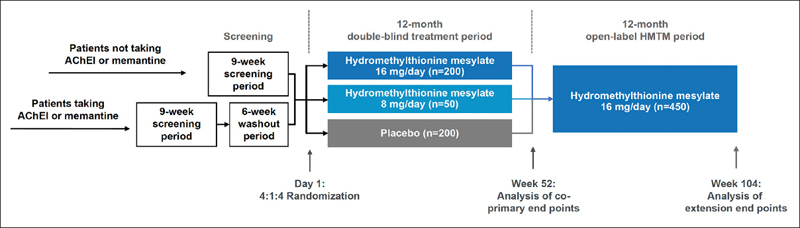
LUCIDITY trial design

The study design is summarized in Figure 4. Following screening, eligible participants are randomized at baseline in a 4:1:4 ratio to receive hydromethylthionine mesylate 16 mg/day, hydromethylthionine mesylate 8 mg/day, or placebo. Following completion of the 52-week double-blind treatment period, all participants continue open-label treatment with hydromethylthionine mesylate 16 mg/day for a further 52 weeks. Participants who were randomized to start on HMTM are considered early-starters; patients who begin on placebo and switch to HMTM at week 52 are considered delayed starters. Unlike a traditional delayed start design, in which participants remain blinded to treatment throughout the trial, treatment during weeks 52 to 104 is open-label, although participants and the site study staff remain blinded to prior treatment assignment. Randomization was stratified by severity (three levels: MMSE 16–19, 20–25, or 26–27, with a target ratio of approximately 2:3:1), prior use of symptomatic treatments (AChEIs and/or memantine — two levels: prior use or none), and region (two levels: Canada/USA or UK/Europe). To achieve this target, enrolment was monitored and controlled at the site level for high recruiting sites and capped as needed at the study level. Patients who dropped out after randomization were not replaced, but participants were encouraged to continue with study visits off treatment until the scheduled completion of the double-blind treatment period (Visit 7). Only participants who continued in the study and receive hydromethylthionine mesylate treatment up to and including the last visit (Visit 10) without the addition of concomitant AChEIs and/or memantine were eligible for continued treatment in a subsequent expanded access program.

### Study Drug and Placebo Formulation

The active and placebo treatment formulations are tablets that look visually the same. Hydromethylthionine mesylate can cause variable urinary discoloration. Therefore, to maintain blinding, the placebo group receives tablets containing a urinary discolourant (MTC, 4 mg) included among blank tablets containing only excipients on a varying schedule with an average frequency of 2/week.

### Inclusion and Exclusion Criteria

Participants had to be aged less than 90 years and meet the diagnostic criteria for probable AD or MCI-AD and must not have been taking an AChEI or memantine, for at least 60 days at Baseline. They must have been community-dwelling, have a mini mental state exam (MMSE) score of 16–27, and with functional impairment as evidenced by a Clinical Dementia Dating (CDR) stage of 0.5 to 2 at screening. Patients must also have had a positive amyloid PET scan. All patients must also have at least one study partner, consenting to their own participation; study partners can be changed so long as they have sufficient contact to complete outcome and safety assessments meaningfully and verify compliance with trial treatment.

Patients were excluded from the study if they have a substantial CNS cause for MCI or dementia other than AD, including significant vascular pathology seen on brain MRI. Other exclusions include severe, unstable, or poorly controlled medical or psychiatric conditions; pregnancy or breastfeeding; contra-indications or previous adverse reactions to MT or excipients; and involvement in another clinical trial or potential for lack of compliance as judged by the investigator. Stable doses of antipsychotics and antidepressants are permitted. Patients with glucose-6-phosphate dehydrogenase (G6PD) deficiency or taking medications with warnings or cautions about methaemoglobinemia are also excluded. Other pharmacologic agents that could affect cognition or pose undue risk are also excluded.

### Recruitment and Consent Procedures

Patients were recruited from memory clinics, outpatient clinics, or other components of specialist neurology, psychiatric, or geriatric medicine services. Where possible, fully informed, written consent was obtained from the patient. If the patient had reduced decision-making capacity, agreement to participate in the study is obtained to the patient’s best level of understanding, supported by consent on the patient’s behalf by a legally authorized representative.

### Assessments

The screening period is up to 9 weeks for participants who are not receiving an AChEI and/or memantine. For participants on AChEI and/or memantine who agree to discontinue, it may be extended for up to a further 6 weeks to allow for wash out. Eight post-Baseline visits are scheduled: five during the double-blind treatment period (Visit 3 for safety assessment; and Visits 4, 5, 6, and 7 at intervals of 3 months for assessments of efficacy, safety, and MRI) and three during the open-label phase. Visit 8, 4 weeks after commencing the open-label phase, is to assess safety; Visits 9 and 10 at intervals of 6 months are for efficacy and safety assessments, with brain imaging only at Visit 10 (Table 1).

**Table 1:** Schedule of post-screening assessments

**Visit (Week)**	**Baseline**		**Double-blind treatment period**					**Open-Label, Delayed-Start phase**			
			**3 (4)**	**4 (13)**	**5 (26)**	**6 (39)**	**7 (52)**		**8 (56)**	**9 (78)**	**10 (104)**
	**Pre Dose**	**Post Dose**					**Pre Dose**	**Post Dose**			
Randomization	X										
Dispense study											
Drug	X			X	X	X	X			X	
ADAS-Cog_13_	X			X	X	X	X			X	X
ADCS ADL_23_	X			X	X	X	X			X	X
Adverse events	X	X	X	X	X	X	X	X	X	X	X
MRI				X	X	X	X			X	X
^18^F-FDG-PET							X				X
Concomitant											
Medication	X		X	X	X	X	X		X	X	X
Physical/neurological											
Examination	X	X	X	X	X	X	X	X		X	X
Ophthalmological examination	X						X				X
Clinical laboratory testing (blood)	X		X	X	X	X	X			X	X
Pregnancy test (blood)	X		X	X	X	X	X			X	X
Vital signs	X	X	X	X	X	X	X	X		X	X
Study drug											
Compliance			X	X	X	X	X		X	X	X
MT concentration (blood)	X	X	X				X	X			X
*APOE* genotype (blood)	X										
MMSE							X				X
CDR							X				X

Abbreviations: *APOE* = apolipoprotein E; ADAS-Cog_13_ = Alzheimer’s Disease Assessment Scale-Cognitive subscale (13-item), ADAS-Cog_11_, used in analyses, is contained in ADAS-Cog_13_; ADCS-ADL_23_ = Alzheimer’s Disease Cooperative Study — Activities of Daily Living (23-item); CDR = Clinical Dementia Rating; ^18^F-FDG-PET = ^18^F-fluorodeoxyglucose-positron emission tomography; MMSE = Mini-Mental State Examination; MRI = magnetic resonance imaging; MT = methylthioninium.

At Visits 2, 3, 7 and 10, timed morning blood samples are collected for determination of plasma level of the drug. Samples are collected prior to dosing and then at 1, 2, and 4 hours post dose. A single blood sample for apolipoprotein E (APOE) genotyping is obtained from participants who provide informed consent at any time after eligibility determination and prior to Visit 7. Blood may also be analyzed for other biomarkers for possible future research related to determination of potential biomarker predictors or surrogates for treatment response, to be described in a separate protocol.

### Safety and Tolerability

All safety assessments are performed during Screening to assess subject eligibility, by an independent qualified medical assessor not involved in efficacy assessments. For the enrolled participants, safety assessments are undertaken at Baseline and at each clinic visit after 4, 13, 26, 39, and 52 weeks during the double-blind treatment period, as well as after 56 (telephone assessment, or on-site in UK), 78, and 104 weeks in the open-label, delayed-start phase; when needed to follow up on a treatment-emergent adverse event (AE); and upon early termination (Table 1). Patients are followed as needed for the resolution or stabilization of any AE, consistent with the investigator’s medical judgement.

### Primary Efficacy End Points

The co-primary end points of the LUCIDITY trial will be assessed in participants taking 16 mg/day hydromethylthionine mesylate, and compared with participants taking placebo. The co-primary end points are change from baseline to Week 52 in cognitive function measured by ADAS-Cog_11_, and functional abilities measured by ADCS-ADL_23_.

### Secondary Efficacy End Points

Secondary end points to be assessed in participants taking placebo compared to participants taking 8 mg/day hydromethylthionine mesylate include:

1. Change from baseline to Week 52 in cognitive function measured by ADAS-Cog_11_, and functional abilities measured by ADCS-ADL_23_.

Secondary end points to be assessed in participants taking placebo compared separately to participants taking 8 mg/day or 16 mg/day hydromethylthionine mesylate include change from baseline to Week 52 in:

1. Cognitive and functional abilities, measured by MMSE and CDR
2. Whole brain, parietal, and temporal lobe volume, measured by MRI; the annualized rate of atrophy from baseline to Week 52 will be quantified using the Boundary Shift Integral (BSI)
3. Brain metabolic function, measured by ^18^F-fluorodeoxyglucose positron emission tomography (^18^F-FDG-PET) change in temporal lobe Standardized Uptake Value Ratio (SUVR) (normalized to pons); this analysis is restricted to participants with a CDR score of 0.5 at Screening, if a predefined threshold is reached for a sufficient number of participants providing data.

Secondary end points to be assessed over the open-label delayed-start period (Week 52 to Week 104) will compare participants originally randomized to placebo (delayed starters) with participants originally randomized to either dose of hydromethylthionine mesylate (early starters) include:

1. Cognitive function measured by change from Week 52 to Week 104 in ADAS-Cog_11_.

### Exploratory End Points

1. TauRx Composite Scale is a new composite designed to be sensitive to decline in early AD constructed on the basis of data available from completed TauRx Phase 3 trials and from Alzheimer’s Disease Neuroimaging Initiative data. The scale consists of cognitive subdomains (orientation, constructional praxis, word recall, assessor’s rating of subject’s speech, and assessor’s rating of subject’s comprehension) from the standard ADAS-Cog_13_, and functional items (use of telephone, keeping appointments, cooking and preparation of meals, and cleaning dishes) from the ADCS-ADL_23_. Scores range from 0–48, with lower scores indicating greater impairment.
2. Change from Week 52 to Week 104 in ADCS-ADL_23_, brain atrophy (MRI), and brain metabolic function (^18^F-FDG-PET), comparing delayed starters with early starters.
3. *APOE* genotype influence on primary and secondary outcomes.
4. Population pharmacokinetic (PK) analyses will be performed to estimate exposure in each subject for use in the evaluation of exposure-response relationships.

### Statistical Analysis

Sample size estimations to achieve 90% power (two-sided alpha = 0.05) to detect a difference between hydromethylthionine mesylate 16 mg/day and placebo, the primary treatment group comparison in the double-blind treatment period, have been performed for the two co-primary clinical end points, assuming a withdrawal rate of 20% to 25%. The study sample size of approximately 450 is based on the ADCS-ADL_23_, which has a larger standard deviation (SD) than ADAS-Cog_11_. Based on an estimated decline in ADCS-ADL_23_ over 52 weeks in the control arm of 7.7 units with an estimated SD of 8.5 units, the study will have >90% power to detect a reduction in decline of 3.4 units or more. The 3.4-unit effect size is derived from an estimated treatment effect of 5.0 ± 1.6 (mean ± standard error) units in the completed hydromethylthionine mesylate studies. Based on an estimated decline of 6.5 units in ADAS-Cog_11_ over 52 weeks with an estimated SD of 5.9 units, 200 participants per treatment arm provide >90% power to detect a reduction in decline of 2.6 units or more, provided by a conservative value of the estimated treatment effect based on pooled completed Phase 3 studies of −5.2 ± 1.3 (mean ± standard error) units in completed hydromethylthionine mesylate studies.

With 200 participants randomized per arm to the primary comparison in the double-blind treatment period, 160 to 170 participants per arm will enter the open-label, delayed-start treatment phase, assuming the 20%–25% dropout rates mentioned above. Assuming a further 10% dropout during the delayed-start phase, the key secondary analysis to demonstrate disease modification by comparing early to late starters using a noninferiority margin of −2 ADAS-Cog_11_ units has approximately 80% power.

The primary analysis will be performed using the intent-to-treat (ITT) and efficacy modified intent-to-treat (E-MITT) populations. The ITT population will include all randomised participants. The E-MITT population will include all randomized participants who took at least one dose of study drug and have a baseline and a valid postbaseline efficacy assessment. The global null hypotheses are as follows:

- H01: There is no difference in the change in ADAS-Cog_11_ between the hydromethylthionine mesylate 16 mg/day and placebo groups from baseline to Week 52.
- H02: There is no difference in the change in ADCS-ADL_23_ between the hydromethylthionine mesylate 16 mg/day and placebo groups from baseline to Week 52.

The global null versus alternative primary efficacy hypotheses is a Union-Intersection Test which requires both the co-primary end points to show statistical significance at the 5% two-sided level of significance, for the global null hypothesis to be rejected.

### Evolution of Protocol Design

The LUCIDITY trial protocol has had 3 major revisions motivated by changing regulatory expectations for AD therapies and emerging data. As initially conceived in 2017 (Version 1.0, August 2017), a limited study was intended to confirm pharmacological activity of the 8 mg/day dose over 6 months in a population of 180 participants meeting diagnostic criteria for early AD, using change in FDG-PET as the primary outcome. At that time, it was envisaged that clinical end points would be assessed in a later larger study in mild to moderate AD. The first revision was in response to draft guidances issued by the FDA in February 2018 and by the European Medicines Agency (EMA) in March 2018 (28, 29) indicating that a single trial could form the basis for regulatory approval in early AD on the basis of a statistically significant benefit with respect to placebo on a single composite clinical outcome scale comprising cognitive and functional elements. Accordingly, the trial was enlarged to 375 participants and lengthened to 9 months, with the intention of using a composite scale developed by TauRx based on the items found to be most sensitive and discriminatory from ADAS-Cog_11_ and ADCS-ADL_23_ scales using data from the completed trials. In light of the emerging exposure-response data summarized above, a dose of 16 mg/day was added to the design. Scientific advice from the EMA in May 2019 indicated that the scope of the approval would be restricted to early AD if the trial were successful. Since hydromethylthionine mesylate has clinically relevant pharmacological activity over AD severity ranging from early to moderate disease (21), the study was changed to a more conventional design with co-primary cognitive (ADAS-Cog_11_) and functional (ADCS-ADL_23_) end points. This entailed a further enlargement to 450 participants in the amended version of the protocol and a longer duration of the double-blind, placebo-controlled treatment phase to 12 months to ensure adequate power. The primary focus of the study was also changed to the 16 mg/day dose. The basic structure of the final amended design (Version 5.0) agreed upon with the EMA was for a double-blind, placebo-controlled treatment period of 12 months, followed by a modified delayed-start open-label extension period of 12 months in which participants, initially randomized to placebo, were switched to 16 mg/day to approximate a delayed-start design to investigate disease-modifying potential. Further modifications were introduced in October 2020 to allow for the impact of COVID-19. The statistical analysis plan was adjusted to accommodate these changes, and the current version is described briefly above. The study was powered on the basis of the primary comparison, 16 mg/day and placebo. A small 8 mg/day arm was retained in a 1:4 ratio with 16 mg/day and placebo to provide a bridge to prior completed trials and to include participants randomised to earlier versions of the protocol receiving this lower dose.

## Results

The study recruited 20% over target in April 2021 due to a late surge in screening enrollments by sites. The target MMSE ratio was achieved. All participants are expected to have completed the blinded phase by the end of March 2022, and top-line results are due mid-2022. Currently, 20% of the participants have terminated early from the double-blind phase. Fewer than 3% of visits have been impacted by COVID-19, and this does not appear to have affected the validity of the trial. A sufficient number of participants with CDR 0.5 have ^18^F-FDG-PET data available to enable analysis of this end point.

## Discussion

Dose selection presents a major challenge in all development programs aiming to develop a disease-modifying treatment for AD. In the case of hydromethylthionine mesylate, selection of 16 mg/day as the optimal dose for treatment of AD was based on a population pharmacokinetic (PK) study using data from over a thousand participants from two Phase 3 trials. From this it became evident that the 8 mg/day dose, which had been intended as an inactive control, had clinically relevant pharmacological activity in the majority of patients and that substantially higher doses did not produce a larger treatment effect. The 16 mg/day dose was identified from the PK data as the minimum required to ensure that all patients would have steady state plasma levels above the therapeutic threshold identified for all patients receiving hydromethylthionine mesylate, the majority of whom were receiving the drug as add-on to standard symptomatic treatments for AD.

The regulatory landscape for pivotal trials in AD has evolved over the period between initial trial design and imminent completion of the LUCIDITY study, culminating in the FDA and EMA Guidances of 2018 (28, 29). It was generally understood until that time that two independent trials were required to show benefit with respect to placebo on cognitive and functional co-primary outcomes. The 2018 Guidances acknowledged that AD is a continuum with a prolonged preclinical phase during which there is progressive development of the underlying brain pathology without manifesting clinically. Furthermore, it was recognized that benefit on functional outcomes may not be demonstrable until later phases of the disease. There was also an acceptance that treatment needed to be initiated early in the disease course to have the best hope of arresting or delaying its inexorable progression. Accordingly, the Guidances proposed that biomarker outcomes were more appropriate for the preclinical phase of the disease. However, there was also a requirement that the actual clinical benefit be demonstrable after longer treatment, or later in the course of the disease. For early AD, it was proposed that a single composite clinical outcome combining cognitive and functional items in a single trial could provide an acceptable basis for initial product approval. The requirement for approval of treatments targeting the mild/moderate stages of the disease remained demonstration of benefit on co-primary cognitive and functional outcomes.

The recent experience with aducanumab has provided a valuable indication of how the 2018 Guidances might affect approval of hydromethylthionine mesylate. A problem with biomarker-based outcomes is that there is no general agreement as to what constitutes a valid surrogate biomarker. Indeed, the problem may be circular, in that until a treatment has been shown to have clinical efficacy which correlates with a biomarker outcome, it is difficult to know what surrogate end point might predict future clinical benefit. The FDA has taken the view that change in the amyloid load in the brain is “reasonably likely to predict clinical benefit”, largely on theoretical grounds. It is on this basis that aducanumab was granted accelerated approval by the FDA (5). However, there is no strong evidence that a reduction in amyloid load is indeed likely to predict clinical benefit, since there is little evidence for a correlation between amyloid load and clinical severity (30–32). This was the view of the EMA, which has not approved aducanumab (33), and it appears unlikely the product will be approved in Japan (34). Likewise, in the absence of evidence of clinical efficacy and safety, the Centers for Medicare & Medicaid Services (CMS), which regulates Medicare coverage in the US, will reimburse aducanumab and related amyloid infusion therapies only in clinical trials aiming to confirm its clinical efficacy (35). The CMS national coverage determination (NCD) would not apply to hydromethylthionine mesylate for two reasons. First, hydromethylthionine mesylate is a small molecule with a mechanism of action targeting tau aggregation, whereas the NCD is specific to monoclonal antibodies directed against amyloid. Second, CMS NCD does not apply to Medicare Part D. Since hydromethylthionine mesylate is delivered orally it would be covered under Medicare Part D, as opposed to the intravenous delivery of aducanumab, which is covered by Medicare Part B.

Since there is evidence that hydromethylthionine mesylate has clinically relevant pharmacological activity over AD severity ranging from mild to moderate dementia, the final design of the LUCIDITY trial agreed with the EMA is unique. This study aims to demonstrate cognitive and functional benefit as co-primary outcomes using well-established scales. However, the design differs in several respects from recent trials targeting the amyloid pathology of AD. The first is the relatively short duration of 12 months for the double-blind placebo-controlled phase. The amyloid-based trials in mild and mild/moderate AD have typically been conducted over 18 months. Since the treatment effect size seen with these approaches is less than the equivalent of −1.5 ADAS-Cog units over 18 months, a long study duration is required to achieve adequate separation between placebo and the active drug. As shown in posthoc analyses, the exposure-dependent treatment effect sizes seen in the completed hydromethylthionine mesylate trials are in the range of 4–5 units over 12 months on both the ADAS-Cog_11_ and ADCS-ADL_23_ scales. A further consideration is that there is evidence of biased withdrawal of participants with moderate disease randomized to placebo over periods longer than 6 months (17). A closely linked difference is the study size. Given the typical within-trial standard deviations on the cognitive (6.43 [95% CI 5.74, 7.13]) and functional (9.94 [(95% CI 9.26, 10.61]) scales determined from the meta-analysis summarized earlier, it is necessary for the study to be large to ensure adequate power to detect a small treatment effect. For the amyloid trials, study sizes have been on the order of 1,000 participants. For the treatment effect sizes seen with hydromethylthionine mesylate, adequate power can be achieved in a study enrolling approximately 450 participants.

A major differentiator between the LUCIDITY study and the majority of other disease-modifying trials conducted to date is the requirement for participants not to be currently taking acetyl-cholinesterase inhibitors or memantine. The earlier hydromethylthionine mesylate trials permitted participants to continue taking these drugs, provided the pretrial dosing regime was maintained with no changes planned during the study. However, as reported in several publications, the treatment effect of hydromethylthionine mesylate is reduced by about half when it is given as an add-on to symptomatic treatments for AD. Therefore, although the trial could have been conducted as an add-on to these treatments, this would have required a study with twice the number of participants or a longer duration or a combination of both. We have elected to conduct the trial in the most efficient manner possible to minimize both the duration and number of participants randomized to placebo. Accordingly, participants are required either to be treatment-naïve or to have washed out from symptomatic treatments.

A further differentiator from other recent trials is the use of whole-brain volume as the principal biomarker outcome. There is good evidence linking tau aggregation pathology with brain atrophy (36–38). Furthermore, it is generally accepted that accelerated brain atrophy is a hallmark of the pathology of the disease. In the case of hydromethylthionine mesylate, there is good evidence of an exposure-dependent reduction in the rate of progression of brain atrophy in posthoc analyses (21). The estimated power for detecting a similar effect in the LUCIDITY trial is similar to that for the clinical outcomes.

Another difference with regard to the earlier hydromethylthionine mesylate trials has been the inclusion of a requirement for a positive amyloid-PET scan. The pharmacological activity of hydromethylthionine mesylate does not require the presence of amyloid pathology since this was not a requirement in the completed Phase 3 trials in which exposure-dependent benefit was shown. The main reason for including this requirement has been the trend in the field to classify dementia on the basis of the presence or absence of amyloid pathology (39, 40). This approach suffers from the need to ascribe etiological, diagnostic, and ultimately therapeutic primacy to amyloid. However, a preliminary report has found no difference in the ultrastructure of the pathological tau filaments isolated from AD and Primary Age-Related Tauopathy (41). Nevertheless, we have elected to conform to the prevailing opinion in this regard to minimize subsequent controversy over diagnosis and disease definition in the present study.

The availability of a drug with the clinical profile of hydromethylthionine mesylate could drive change in the clinical management of AD. Dementia is widely under-diagnosed, partially due to the lack of treatment options (3, 42, 43). The availability of a disease-modifying treatment would motivate earlier diagnosis by patients, caregivers, health care providers, and payers. Governments have recognized this and, for example, the Governor of Pennsylvania recently signed the House Bill 1082, requiring the Department of Health to build a toolkit and education program for primary care providers, that provides details on the diagnosis, treatment, and prevention of AD.

In summary, the ongoing LUCIDITY study aims to evaluate the efficacy and safety of hydromethylthionine mesylate at a dose of 16 mg/day, with the aim of demonstrating statistically significant benefit with respect to placebo on co-primary cognitive and functional clinical end points. If the profile seen in the completed hydromethylthionine mesylate trials in participants with therapeutic levels of exposure at the 8 mg/day dose is confirmed in the LUCIDITY trial, hydromethylthionine mesylate has the potential to contribute positively to the management of AD. Top-line results of the double-blind phase are expected in mid-2022.

## Notes

*Ethical standard:* The protocol was approved by an institutional review board or independent ethics committee at each site, and is conducted in accordance with the Declaration of Helsinki and the International Conference on Harmonization and Good Clinical Practice guidelines.
